# Ducks as Sentinels for Avian Influenza in Wild Birds

**DOI:** 10.3201/eid1510.090439

**Published:** 2009-10

**Authors:** Anja Globig, Anette Baumer, Sandra Revilla-Fernández, Martin Beer, Eveline Wodak, Maria Fink, Norbert Greber, Timm C. Harder, Hendrik Wilking, Iris Brunhart, Doris Matthes, Ulf Kraatz, Peter Strunk, Wolfgang Fiedler, Sasan R. Fereidouni, Christoph Staubach, Franz J. Conraths, Chris Griot, Thomas C. Mettenleiter, Katharina D.C. Stärk

**Affiliations:** Friedrich-Loeffler-Institute, Greifswald-Insel Riems, Germany (A. Globig, M. Beer, T.C. Harder, H. Wilking, U. Kraatz, P. Strunk, S.R. Fereidouni, C. Staubach, F.J. Conraths, T.C. Mettenleiter); Institute of Virology and Immunoprophylaxis, Mittelhäusern, Switzerland (A. Baumer, C. Griot); Austrian Agency for Health and Food Safety, Mödling, Austria (S. Revilla-Fernández, E. Wodak, M. Fink); State of Vorarlberg Veterinary Directorate, Vorarlberg, Austria (N. Greber); Bird Ringing Centre, Radolfzell, Germany (D. Matthes, W. Fiedler); Federal Veterinary Office, Bern, Switzerland (I. Brunhart); Royal Veterinary College, London, UK (K.D.C. Stärk)

**Keywords:** sentinel, avian influenza, active surveillance, wild birds, influenza, viruses, dispatch

## Abstract

To determine the effectiveness of ducks as sentinels for avian influenza virus (AIV) infection, we placed mallards in contact with wild birds at resting sites in Germany, Austria, and Switzerland. Infections of sentinel birds with different AIV subtypes confirmed the value of such surveillance for AIV monitoring.

As a consequence of infections of wild birds and poultry with highly pathogenic avian influenza virus (HPAIV) subtype H5N1, surveillance of wild birds for avian influenza viruses (AIVs) has intensified in Europe since 2005. Reporting of results is compulsory in the European Union (*1*,*2*). HPAIV surveillance includes investigation of dead or sick wild birds (*3*) with the aim of early detection of HPAIV (H5N1) complemented by sampling of healthy wild birds to monitor for low pathogenicity (LP) AIV strains (*4*). Previously, sentinel birds were used successfully to obtain information about AIV subtypes circulating in wild birds (*5*), but results of those studies are now outdated. Also, the effectiveness of sentinel birds has not yet been documented for AIV strains that emerged during the past decade.

We evaluated a sentinel approach to monitor the prevalence of HPAIV and LPAIV within an ecosystem, obtain information about seroconversion and duration of immunity after infection with AIV, and serve as an early warning system for the introduction of HPAIV (H5N1) and other notifiable AIVs (subtypes H5 and H7) to wild bird populations. Here we summarize results from a 2-year period of 3 international sentinel projects ongoing since 2006.

## The Study

**Figure 1:**
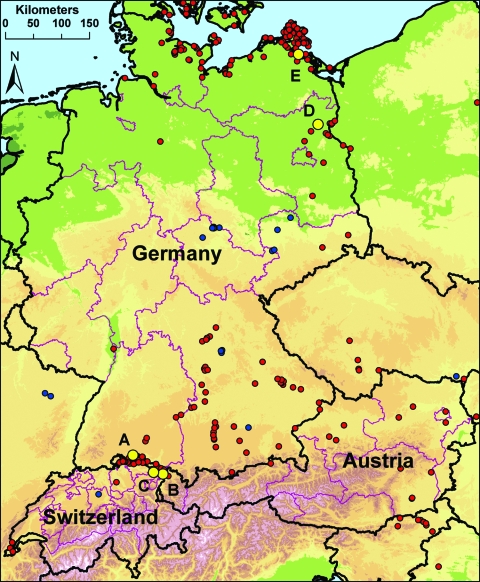
Locations of sentinel duck flocks at 5 locations in Germany, Switzerland, and Austria. A–C) Sites at Lake Constance: Radolfzell, Germany (A); Bregenz-Thal, Austria (B); and Altenrhein, Switzerland (C). D–E) Additional sentinel stations at Lake Felchow, Brandenburg, Germany (D), and Isle of Koos, Mecklenburg–Western Pomerania, Germany (E). Yellow dots mark the location of sentinel stations. Red dots mark detections of highly pathogenic avian influenza virus (HPAIV) (H5N1) in dead wild birds in 2006, and blue dots in 2007. In 2008, HPAIV (H5N1) was not found in dead wild birds in any of the 3 countries but was found in a live pochard (*Aythya ferina*) from Switzerland/Lake Sempach (blue dot in Switzerland).

In 2006, multiple introductions and spread of HPAIV (H5N1) occurred in Europe, including the wetlands in Austria, Germany, and Switzerland (*3*,*4*,*6*,*7*). For our study, we selected 5 locations with substantial and heterogeneous wild bird populations on the basis of HPAIV (H5N1) subtype detected during 2006. Sentinel stations were located around Lake Constance and in 2 other wetlands in Germany. The sentinel flocks at Lake Constance were situated in 1) Radolfzell (Möggingen), Germany (47°45′58′′N, 8°59′45′′E); 2) Altenrhein, Switzerland (47°29′25′′N, 9°32′45′′E); and 3) Bregenz-Thal (Rheindelta), Austria (47°30′60′′N, 9°38′55′′E). The 2 other stations were situated 4) on the Isle of Koos close to the Island of Rügen in Mecklenburg–Western Pomerania, Germany (54°10′13′′N, 13°24′11′′E) and 5) near the Oder Valley at Lake Felchow (Brandenburg), Germany, (53°03′09′′N, 14°08′06′′E) (Figure 1). After their wing feathers were clipped, 10–20 hand-bred adult mallard ducks (*Anas plathyrhynchos*) <1 year of age were placed in pens in natural water bodies, allowing continuous direct contact with wild water birds as previously described (*5*). Cloacal and oropharyngeal swabs and blood were taken from the mallards and tested negatively by using 1-step TaqMan real-time reverse transcription–PCR (RT-PCR) or competitive nucleoprotein antibody ELISA (cNP ELISA) (ID-Vet, Montpellier, France; Anigen, MegaCorGmbH, Hörbanz, Austria) before their use as sentinels.

At all sentinel stations in Germany, we collected oropharyngeal and cloacal swab samples from the sentinels every 14 days starting in autumn 2006. Sampling at the station in Austria started in February 2007 and at the station in Switzerland in October 2007. Laboratory tests were conducted in accordance with the Diagnostic Manual for Avian Influenza of the European Union (*8*). RNA was isolated from swabs by using viral RNA kits (QIAGEN, Hilden, Germany; Macherey-Nagel, Oensingen, Switzerland) and analyzed by real-time RT-PCR for influenza virus matrix (M) or nucleocapsid protein (NP) gene fragments. In positive samples, H5-, N1- and H7-specific real-time RT-PCRs were used to identify or exclude respective subtypes (*9*,*10*). H5 and H7 isolates were pathotyped following the European Union directive (*8*). Direct hemagglutinin (HA) typing or sequencing of positive samples was carried out as previously described (*7*,*11*–*13*). The neuraminidase (NA) subtype was identified molecularly, by following the method of Fereidouni et al. (*14*). Simultaneously, we attempted virus isolation in embryonated chicken eggs from positive samples (*8*).

**Figure 2:**
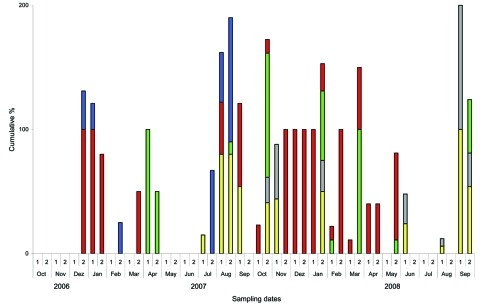
Months with positive results for sentinel birds over a 2-year period at 5 locations in Germany, Switzerland, and Austria. Sites at Lake Constance: Radolfzell, Germany (yellow); Bregenz-Thal, Austria (gray); and Altenrhein, Switzerland (green). Additional sentinel stations at Lake Felchow, Brandenburg, Germany (red), and Isle of Koos, Mecklenburg–Western Pomerania, Germany (blue). Bars indicate the cumulative percentage of sentinel birds tested positive at each of the 5 locations at the time of sampling (maximum 500% at all 5 stations). For example, in December 2006, all sentinel ducks at station 4, but only 30% of sentinels at station 5, were positive at the date of sampling. 1, days 1–15 of month; 2, day 16 through end of month.

From October 2006 through September 2008, at least 23 specifiable AIV infections were detected at the sentinel stations by the fortnightly swabbing. After initial AIV introduction, virus was excreted during the following 1–3 sampling dates (Figure 2). All ducks at all sites tested positive at least once. Infections caused by AIV of 8 HA subtypes, including H5 and H7, and 6 NA subtypes, were found in clinically healthy sentinel birds (Tables 1, 2). Viral RNA and, in 44% of AIV cases, infectious virus also were recovered both from cloacal and oropharyngeal swabs. AIVs were subtyped as H1N1, H1Nx, H2N2, H2N5, H3N2, H3Nx, H3N8, H4N6, H5Nx, H6N5, H6N8, H7N3, H7Nx, and H9N2. Cycle threshold values ranged from 24 to 40. Pathotyping of H5 and H7 subtypes showed the exclusive presence of LP viruses. Additional AIVs were detected but could neither be isolated nor sequenced for subtype identification because of low loads of viral RNA. However, we did not detect H5, N1, and H7 subtypes by using real-time RT-PCR. Infections occurred most frequently from August through January (Figure 2; Table 1). Reinfection of the sentinels with the same subtype occurred in 2 of the sentinel flocks in Germany (Table 1).

**Table 1:** Detection of AIV by sampling of sentinel mallard ducks (*Anas plathyrhynchos*) at 5 locations in Germany, Switzerland, and Austria*†

Sentinel location	Date sampled	Ct range‡	Duration virus excretion, d	HA subtype	NA subtype	Isolate	Sequence of HA-cleavage site (H5, H7)
Radolfzell, Germany	2007 Aug	29–37	Min 28, max 42	H6	N8	Yes	
	2007 Oct	31–39	Min 14, max 28	H2	N5	Yes	
				H3	N2	Yes	
	2008 Jan	33–38	Punctual	H3	N2	Yes	
	2008 Jun	33–39	Punctual	H3	N2	No	
	2008 Sep	33–39	Min 14, max 28	H3	N8	Yes	
Altenrhein, Switzerland	2007 Oct	27–39	Min 15, max 39	ND§	N2	No	
	2007 Dec	24–37	Punctual	H2	N2	No	
	2008 Aug	29–34	Punctual	H9	N2	No	
Bregenz-Thal, Austria	2007 Apr	28–38	Min 23, max 38	H3	ND¶	No	
	2007 Oct	22–40	Min 12, max 26	H9	N2	Yes	
	2008 Jan	27–38	Min 14, max 42	H1	N1	No	
	2008 Mar	25–35	Min 14, max 42	H7	ND¶	No	PEIPKGR GLF
Lake Felchow, Brandenburg, Germany	2006 Dec	27–35	Min 22, max 36	H6	N2	Yes	
	2007 Jan	29–38	Min 14, max 34	H5	N3?	No	PQRETR GLF
	2007 Mar	35–38	Punctual	H5	ND¶	No	
	2007 Sep	31–39	Punctual	H6	N5	Yes	
	2007 Dec	27–37	Min 42, max 56	H9	ND¶	No	
				H1?	ND¶	No	
				H11?	ND¶	No	
	2008 Feb/Mar	30–38	Min 56, max 70	H9	ND¶	No	
Isle of Koos, Mecklenburg–Western Pomerania, Germany	2006 Dec	29–35	Min 14, max 28	H4	N6	Yes	
	2007 Aug	32–38	Min 35, max 49	H7	N3	Yes	PEIPKGR GLF

*Radolfzell, Altenrhein, and Bregenz-Thal are located along Lake Constance. AIV, avian influenza virus; Ct, cycle threshold value; HA, hemagglutinin; NA, neuraminidase; min, minimum; max, maximum; ND, not determined. †Each sentinel flock comprised 10–20 birds. Only the initial detection of each AIV introduction and determined HA or NA subtypes are presented. ‡Results of matrix and nucleocapsid protein gene fragments by real-time reverse transcription–PCR. §AIV-positive, HA subtype not determined, non-H5, non-H7. ¶AIV-positive, NA subtype not determined, non-N1.

**Table 2:** Frequency of sentinel duck sampling and frequency of AIV detection at 5 locations in Germany, Switzerland, and Austria*

Sentinel location	Investigation period	No. samplings of sentinel flock†	No. AIV detections	AIV subtypes
Radolfzell, Germany	2006 Oct–2008 Sep	53	11	H6N8, H2N5, H3N2, H3N8
Altenrhein, Switzerland	2007 Oct–2008 Sep	24	7	H2N2, H9N2, HxN2
Bregenz-Thal, Austria	2007 Feb–2008 Sep	44	9	H9N2, H3Nx, H1N1, H1Nx, LP H7Nx
Brandenburg, Germany	2006 Oct–2008 May	41	20	H1?, LP H5Nx, H6N2, H6N5, H9Nx, H11?
Mecklenburg–Western Pomerania, Germany	2006 Oct– 2008 Jun	40	6	H4N6, LP H7N3

*Radolfzell, Altenrhein, and Bregenz-Thal are located along Lake Constance. AIV, avian influenza virus; LP, low pathogenicity. †Each flock consisted of 10–20 birds.

Blood samples were collected from the ducks once a month, and serum was tested in a cNP-ELISA after heat inactivation at 56°C for 30 min. After each natural infection, sentinel animals seroconverted, and serum scored positive in the cNP-ELISA within 2–4 weeks. By hemagglutination inhibition test using homosubtypic but not autologous antigen, HA-specific antibodies were detected only rarely and at low titers.

Detection rates of AIV in sentinel ducks were compared with data from monitoring of healthy, trapped wild birds. From October 2006 through September 2008, a total of 1,953 wild birds were investigated for AIV within a radius of 30 km of Lake Constance, resulting in 47 (2.4%) AIV detections of subtypes H3Nx, LP H5N2, H6N8, LP H7Nx, H1N1, HxN1, H1Nx, and H9N2. During January 2007–May 2008, a total of 8 (0.4%) of 2,028 investigated wild bird samples from Brandenburg tested positive (subtypes H3N6, H6). In Mecklenburg–Western Pomerania, 8,066 birds were tested; 23 (0.3%) AIV infections (subtypes H1Nx, LP H5N2, H6Nx, H12Nx, H16Nx) were found.

## Conclusions

In practice, AIV surveillance of live wild birds is difficult and involves substantial labor and costs, particularly for purchase and maintenance of trapping equipment, salary of trapping staff, and laboratory analysis. Trapping of wild birds also can be biased by season and by bird species that are easier to catch. Low proportions of AIV-positive results (<3%) indicate the low cost:benefit ratio of surveillance based on trapping wild birds (*2*). In contrast, our findings demonstrate that the use of sentinel birds in regions with substantial wild bird populations achieves a high rate of AIV detection and, therefore, is an efficient supplement to active AIV monitoring. The detection of different AIVs among the sentinel ducks reflects the natural ecology of AIV at discrete locations. Recently, all duck species, especially dabbling ducks, have been assessed as high-risk species for possibly contributing to the transmission of HPAIV (H5N1) (*15*). Therefore, mallards as sentinel species ensure a high probability of detecting AIV if kept in direct contact with wild water birds. In addition, sites for sentinel stations need to be selected carefully to achieve spatial representation.

Although our study was conducted in areas where HPAIV (H5N1) had circulated in wild birds in 2006, this subtype was not found by screening live wild birds or by using sentinel birds during the study period. Therefore, persistent circulation of HPAIV (H5N1) in the wild bird populations is unlikely for the area of Lake Constance, the coastal area of Mecklenburg–Western Pomerania, and the region of the Oder Valley in Brandenburg. However, because of the limited sample sizes, a low prevalence cannot be excluded. Although HPAIV (H5N1) was found only rarely in apparently healthy birds, e.g., in a pochard (*Aythya ferina*) in Switzerland in 2008 (Figure 1), regular testing of sentinel birds could increase the probability of detecting sporadic transmission of HPAIV in healthy wild water birds even in the absence of detectable deaths.
